# Acquired Reactive Perforating Collagenosis: A Case Report

**DOI:** 10.7759/cureus.13583

**Published:** 2021-02-27

**Authors:** Madiha Eljazouly, Maha Alj, Fatimazahra Chahboun, Hafsa Chahdi, Soumiya Chiheb

**Affiliations:** 1 Dermatology, Cheikh Khalifa International University Hospital, Mohammed VI University of Health Sciences, Casablanca, MAR; 2 Pathology, Cheikh Khalifa International University Hospital, Mohammed VI University of Health Sciences, Casablanca, MAR; 3 Pathology, Military Hospital Mohammed V, Rabat, MAR; 4 Dermatology, Ibn Rochd University Hospital, Casablanca, MAR

**Keywords:** perforating dermatosis, familial reactive perforating collagenosis, acquired perforating collagenosis

## Abstract

Reactive perforating collagenosis (RPC) is a rare form of dermatosis. It forms with perforating folliculitis, Kyrle's disease, and serpiginous perforating elastosis, which is a group of perforating dermatosis. RPC can be hereditary with autosomal dominant transmission or it can be acquired, which is usually observed in diabetics with chronic renal failure. Here we report a new observation in a 72-year-old woman treated by phototherapy with a favorable outcome

## Introduction

Reactive perforating collagenosis (RPC) is a rare form of dermatosis. It was first described in 1967 by Mehregan et al. [1]. It forms with perforating folliculitis, Kyrle's disease, and serpiginous perforating elastosis (EPS), which is a group of perforating dermatosis. It is important to underline the association of these various disorders with many diseases as EPS is associated with pseudoxanthoma elasticum, Down syndrome, osteogenesis imperfecta, Ehlers-Danlos syndrome, Rothmund-Thomson syndrome, Marfan syndrome, and the use of D-penicillamine [2]. RPC can be hereditary with autosomal dominant transmission or it can be acquired, which is commonly associated with diabetes and chronic kidney disease. We report a new observation that illustrates the clinical, pathologic, and histochemical features of acquired RPC (ARPC).

## Case presentation

**Figure 1:**
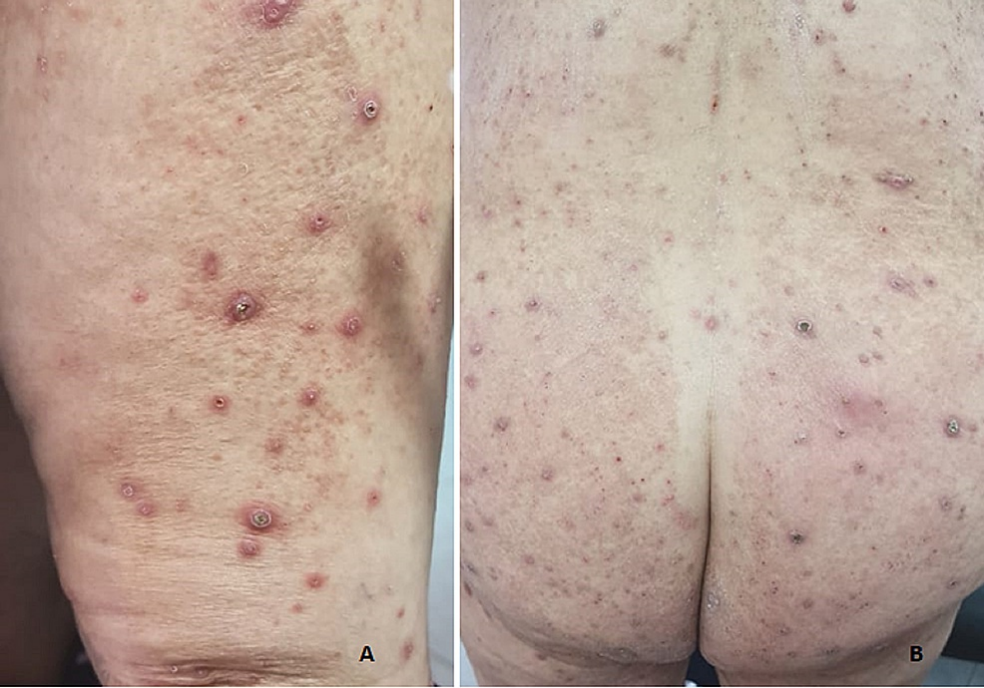
Skin lesions primarly located on the limb (A) and extending to the trunk and back surface (B).

**Figure 2:**
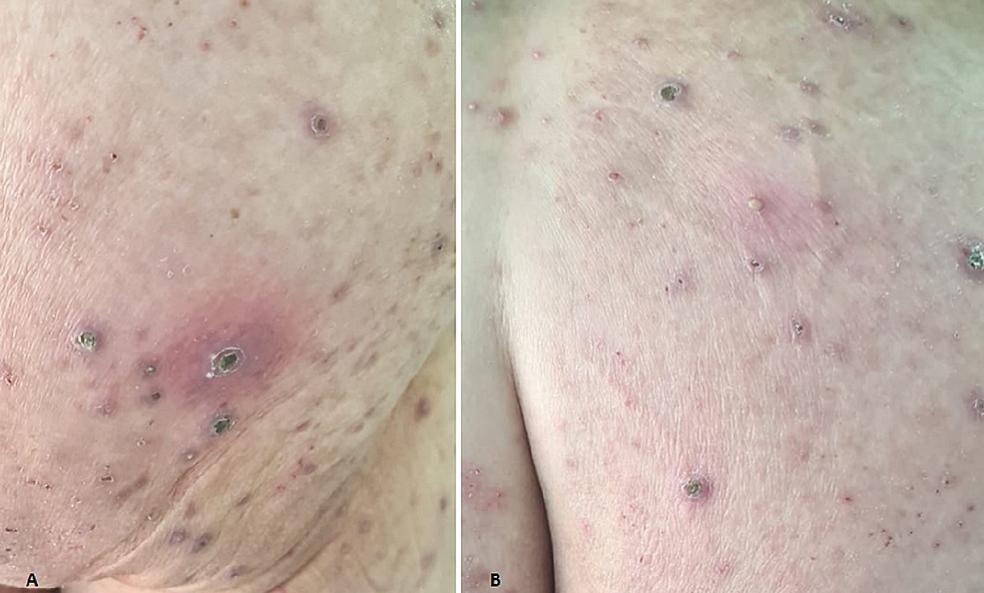
Umbilicated papules and nodules with central keratine plug. (A) Inflammatory pustular lesions with brown crusts and dark scars (B).

**Figure 3:**
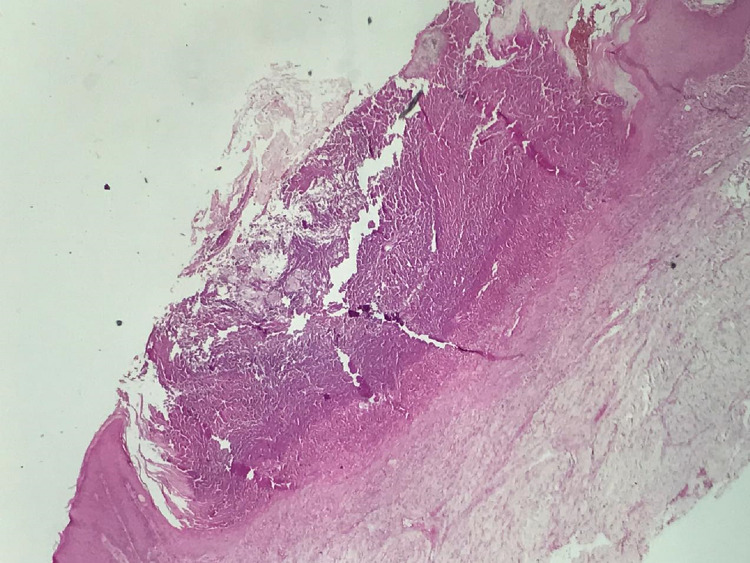
Crateriform epidermal ulceration with acanthosis filled with nuclear debris, keratin, and inflammatory cells (hematoxylin and eosin ×25).

**Figure 4:**
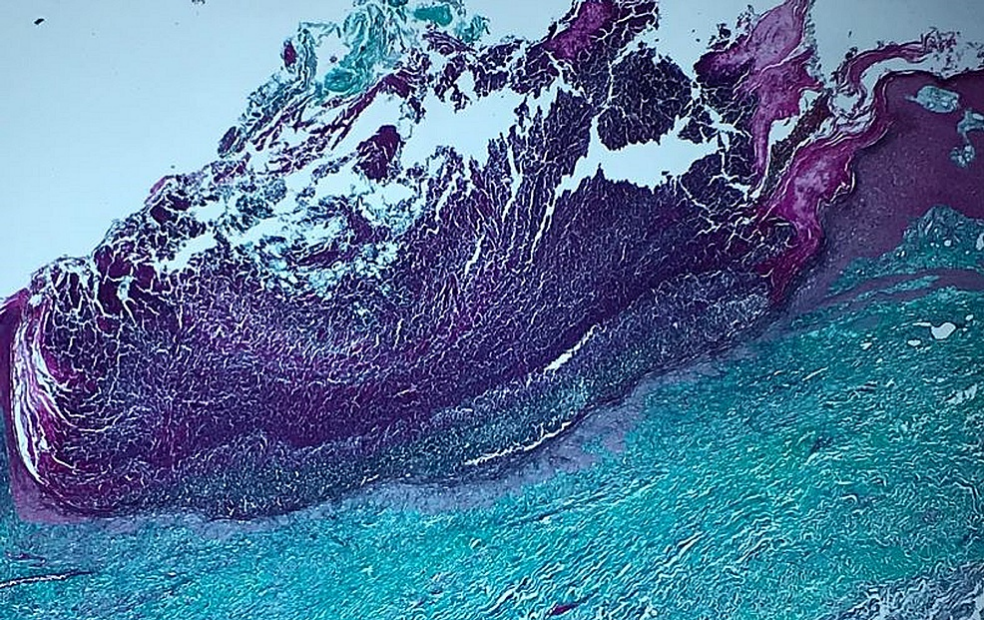
Altered collagen in oblique strands indicating transepidermal elimination (Masson's trichrome staining ×25).

A 72-year-old patient, followed for non-insulin-dependent diabetes with chronic renal failure, presented with generalized chronic pruritus with a very itchy skin lesion that had been evolving for six months and was initially located on the limbs with extension to the back and trunk but sparing the face (Figures 1A, 1B). The clinical examination revealed multiple papulonodular lesions with central umbilicated necrosis and keratinous plug (Figure 2A) associated with pustular lesions and excoriated pigmented atrophic scars (Figure 2B). Histopathology showed degenerated keratin components in the central goblet epidermis (Figure 3). Masson's trichrome staining revealed altered collagen fibers (Figure 4). Peripheral blood cell count and biochemical examination showed an inflammatory syndrome with anemia at 9 g/L serum phosphorus at 58 mg /L. Hepatitis C virus antibodies and thyroid function tests were normal. The ultrasonic examination was normal. The case was diagnosed as ARPC. UVB TL01 phototherapy with dialysis sessions combined with topical corticoids resulted in a regression of pruritus and lesions.

## Discussion

Perforating dermatosis is defined by the transepidermal removal of components of the dermis, in particular collagen and/or elastic fibers. RPC is caused by the removal of damaged collagen fibers. Its hereditary form is characterized by an early onset in childhood or can occur late in childhood at trauma sites, particularly in the arms and hands [3]. The affection can evolve throughout life, with remissions, especially in summer. Family cases are classically reported [4]. Its acquired type occurring in adulthood is the most frequently described form in the literature. It occurs mainly in cases of renal failure and diabetics patients and is more rarely associated with solid tumors, AIDS, lymphomas, and Hodgkin's disease [5]. Sporadic cases have been reported with systemic lupus erythematosus, dermatomyositis, and during treatment with erlotinib [6,7]. However, it should be pointed that some cases of dermatosis can occasionally take on a perforating appearance clinically or histologically, such as keratoacanthoma, foreign body granuloma, amyloidosis, and lichen nitidus. Patterson et al. have identified and classified all of these conditions as atypical perforating dermatosis [8]. For this reason, it is currently recommended that the cases observed in diabetics and renal failure patients be classified as acquired perforating dermatosis (APC) rather than as ARPC [9]. Although the mechanism of atypical perforating dermatosis remains unknown, several authors suggest the role of scratching secondary to pruritus and repeated micro-traumatisms in the triggering of ARPC associated with Hodgkin's lymphoma, solid tumors, thyroid and parathyroid pathology, or infections such as AIDS [10]. In diabetic patients, the abnormal glycation observed after long-term hyperglycemia is the cause of the altered collagen I and III [11]. The diagnosis of ARPC is anatomoclinical. It is commonly manifested by umbilicated papular lesions eventually, and follicular and hyperkeratotic in general. Almost always the lesions are pruritic, and new lesions appear followed by Koebner’s phenomenon. In our patient, a clinical polymorphism between APC and perforating folliculitis was observed, suggesting the possible overlap of perforating dermatosis, which is reported in the literature particularly in patients with chronic renal failure [12,13]. The histological study combined with Masson's trichrome stain shows a dome-shaped lesion with a central crater, focal epidermal ulceration, covered by a hyperkeratotic crust, and penetration of underlying dermal tissues through the dermoepidermal junction. Once again, the overlap noted clinically was also raised histologically by Indian authors with the coexistence of the histological image of ARPC and perforating folliculitis [2,13]. Dermoscopy findings can also contribute to the diagnosis and reveal a red-brown structureless area covered with crusts and scales centrally, surrounded by a white rim, and a reddish inflammatory circle with peripheral vessels [12,14]. The treatment of perforating dermatoses is difficult. There is currently no consensus on treatment. Several therapeutic options have been reported as first-line therapy in ARPC, including topical corticoids, local and systemic retinoids, and UVB-TL01 phototherapy with variable results. Dynamic phototherapy, amitriptyline, and doxycycline have also been the subject of publications [15-17]. Our patient was successfully treated with a combination of UVB phototherapy and dialysis session.

## Conclusions

ARPC is an understood nosological entity, which is frequently accompanied by several systemic diseases including malignant conditions. Thus, thorough paraclinical exploration is necessary to reveal a possible underlying extracutaneous disease.
